# Cardiac and inflammatory biomarker differences in adverse cardiac events after chimeric antigen receptor T-Cell therapy: an exploratory study

**DOI:** 10.1186/s40959-023-00170-5

**Published:** 2023-04-01

**Authors:** Dae Hyun Lee, Sanjay Chandrasekhar, Michael D. Jain, Rahul Mhaskar, Kayla Reid, Sae Bom Lee, Salvatore Corallo, Melanie J. Hidalgo-Vargas, Abhishek Kumar, Julio Chavez, Bijal Shah, Aleksandr Lazaryan, Farhad Khimani, Taiga Nishihori, Christina Bachmeier, Rawan Faramand, Michael G. Fradley, Daniel Jeong, Guilherme H. Oliveira, Frederick L. Locke, Marco L Davila, Mohammed Alomar

**Affiliations:** 1 Division of Cardiovascular Sciences, Morsani College of Medicine, University of South Florida, Tampa, FL USA; 2 Cardio-Oncology Program, H. Lee Moffitt Cancer Center and Research Institute, Tampa, FL USA; 3 Department of Blood and Marrow Transplant and Cellular Immunotherapy, H. Lee Moffitt Cancer Center and Research Institute, Tampa, FL USA; 4 Department of Oncologic Sciences, Morsani College of Medicine, University of South Florida, Tampa, FL USA; 5 Department of Internal Medicine, Morsani College of Medicine, University of South Florida, Tampa, FL USA; 6 Cardio-Oncology Center of Excellence, University of Pennsylvania Perelman School of Medicine, Philadelphia, PA USA; 7 Department of Radiology, H. Lee Moffitt Cancer Center and Research Institute, Tampa, FL USA; 8 Department of Malignant Hematology, H. Lee Moffitt Cancer Center and Research Institute, Tampa, FL USA; 9 Cardio-Oncology Program, H. Lee Moffitt Cancer Center and Research Institute, 12902 USF Magnolia Drive, CSB 3130, Tampa, Florida 33612 USA

**Keywords:** Chimeric antigen receptor T cell, Cardiotoxicity, Cardio-oncology, Cardiac biomarker, Tocilizumab, Inflammatory cytokines

## Abstract

**Background:**

Chimeric antigen receptor T- Cell (CAR-T) immunotherapy has been a breakthrough treatment for various hematological malignancies. However, cardiotoxicities such as new-onset heart failure, arrhythmia, acute coronary syndrome and cardiovascular death occur in 10–15% of patients treated with CAR-T. This study aims to investigate the changes in cardiac and inflammatory biomarkers in CAR-T therapy to determine the role of pro-inflammatory cytokines.

**Methods:**

In this observational study, ninety consecutive patients treated with CAR-T underwent baseline cardiac investigation with electrocardiogram (ECG), transthoracic echocardiogram (TTE), troponin-I, and B-type natriuretic peptide (BNP). Follow-up ECG, troponin-I and BNP were obtained five days post- CAR-T. In a subset of patients (N = 53), serum inflammatory cytokines interleukin (IL)-2, IL-6, IL-15, interferon (IFN)-γ, tumor necrosis factor (TNF)-α, granulocyte-macrophage colony-stimulating factor (GM-CSF), and angiopoietin 1 & 2 were tested serially, including baseline and daily during hospitalization. Adverse cardiac events were defined as new-onset cardiomyopathy/heart failure, acute coronary syndrome, arrhythmia and cardiovascular death.

**Results:**

Eleven patients (12%) had adverse cardiac events (one with new-onset cardiomyopathy and ten with new-onset atrial fibrillation). Adverse cardiac events appear to have occurred among patients with advanced age (77 vs. 66 years; p = 0.002), higher baseline creatinine (0.9 vs. 0.7 mg/dL; 0.007) and higher left atrial volume index (23.9 vs. 16.9mL/m^2^; p = 0.042). Day 5 BNP levels (125 vs. 63pg/mL; p = 0.019), but not troponin-I, were higher in patients with adverse cardiac events, compared to those without. The maximum levels of IL-6 (3855.0 vs. 254.0 pg/mL; p = 0.021), IFN-γ (474.0 vs. 48.8pg/mL; p = 0.006) and IL-15 (70.2 vs. 39.2pg/mL; p = 0.026) were also higher in the adverse cardiac events group. However, cardiac and inflammatory biomarker levels were not associated with cardiac events. Patients who developed cardiac events did not exhibit worse survival compared to patients without cardiac events (Log-rank p = 0.200).

**Conclusion:**

Adverse cardiac events, predominantly atrial fibrillation, occur commonly after CAR-T (12%). The changes in serial inflammatory cytokine after CAR-T in the setting of adverse cardiac events suggests pro-inflammation as a pathophysiology and require further investigation for their role in adverse cardiac events.

**Tweet brief handle:**

CAR-T related Cardiotoxicity has elevated cardiac and inflammatory biomarkers. #CARTCell #CardioOnc #CardioImmunology.

**Supplementary Information:**

The online version contains supplementary material available at 10.1186/s40959-023-00170-5.

## Introduction

Chimeric antigen receptor T- Cell (CAR-T) immunotherapy has been a breakthrough for various CD19 + hematological malignancies, including lymphoma [1–3]. One serious complication of CAR-T therapy is cytokine release syndrome (CRS), caused by an exaggerated immune-system activation leading to fever, hypotension, hypoxia, and end-organ damage [4, 5]. In high-grade CRS, various cardiotoxicities including cardiomyopathy, arrhythmias, and cardiovascular death can occur [6–8].

The pathophysiology of these cardiovascular toxicities is thought to be secondary to CRS and potentiated by both cardiac and non-cardiac risk factors [9, 10]. These include a higher degree of CRS [6, 7, 11], baseline creatinine level [7], troponin-I elevation after CAR-T infusion [6], presence of neurotoxicity [8], and type of CAR-T [12]. Although cardiac events are clinically related to CRS, the data on specific inflammatory cytokine levels in CAR-T- related cardiotoxicities is lacking. The role of cardiac and inflammatory biomarkers in the management of CAR-T associated cardiotoxicity is also not well established.

The purpose of this study was to investigate the changes of cardiac biomarkers and inflammatory cytokines that occur in the setting of adverse cardiac events after CAR-T therapy.

## Method

### Study population

This was an observational study in a single, National Cancer Institute (NCI)-designated academic center (H. Lee Moffitt Cancer Center). This study was approved by the IRB of the University of South Florida (Pro00029257 and Pro00021733). Participating patients provided informed consent for the quantification of inflammatory cytokines. Inclusion criteria included patients diagnosed with B-cell lymphoma (diffuse large B cell lymphoma, mantle cell lymphoma, follicular lymphoma, and B-Cell acute lymphoblastic leukemia) who were treated with four types of CAR-T therapy (Axicabtagene ciloleucel- Yescarta®, tisagenlecleucel- Kymriah®, Brexucabtagene autoleucel- Tecartus® and lisocabtagene maraleucel- Breyanzi®). As a collaborative work between our cardio-oncology team and CAR-T team, we set up standard clinical practice guidelines at our institution beginning October 2020. Consecutive patients undergoing CAR-T therapy from October 2020 until October 2021 underwent baseline and follow-up cardiac biomarkers. This study included a retrospective review clinical data, as summarized in below section. In addition, of the consecutive patients reviewed, there were subset of patients who were enrolled in prospective observational study investigating the validity of daily cytokine monitoring system for CRS.

### Standard clinical management

**Fig. 1:**
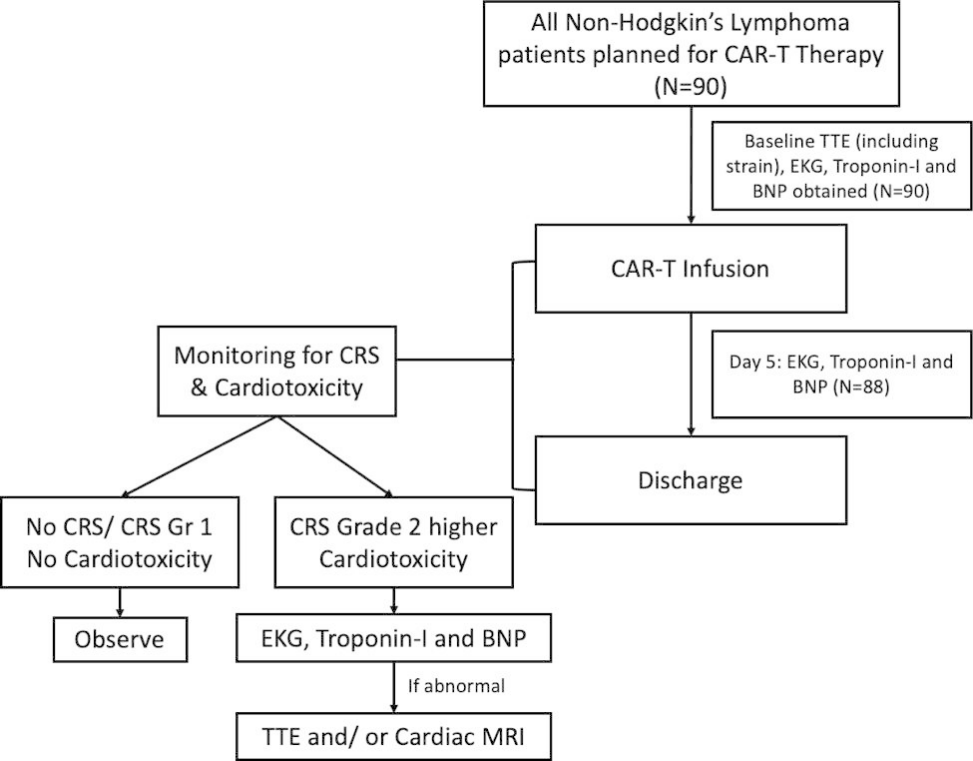
Diagnostic Workflow for CAR-T Patients The standard clinical practice at our institution includes comprehensive cardiac evaluation including transthoracic echocardiogram, electrocardiogram and cardiac biomarkers. During the index hospitalization after CAR-T infusion, close surveillance for adverse cardiac events occurs with follow-up cardiac biomarkers. If a patient develops cytokine release syndrome grade 2 or higher or develop new onset cardiotoxicity, an electrocardiogram and cardiac biomarkers are obtained.

Baseline cardiac investigation at the time of evaluation for CAR-T therapy included electrocardiogram, transthoracic echocardiogram, troponin-I, and B-type natriuretic peptide (BNP). Follow-up ECG, troponin-I, and BNP levels were obtained five days after CAR-T infusion and at the development of CRS Grade ≥2 (fever with hypotension or hypoxia). The CRS grading was based on published guidelines by the American Society for Transplantation and Cellular Therapy (ASTCT) [5]. The cardio-oncology team reviewed the data and evaluated the patient clinically. If screening measures with troponin-I, BNP, or EKG were abnormal, a repeat echocardiogram and subsequent cardiac MRI, if clinically indicated. (Fig. 1) Troponin-I and BNP were considered abnormal if above the reference value (reference value < 0.03ng/mL for troponin I and reference value < 100pg/mL for BNP). EKGs were considered abnormal if there were tachyarrhythmia (atrial fibrillation/ flutter, ventricular tachycardia), bradyarrhythmia (sinus bradycardia, 2nd degree or higher AV block), or ST-T segment changes. Chemotherapy-related cardiac dysfunction (CTRCD) was defined as a new LVEF reduction of ≥10%, LVEF less than 50%, or relative decline of the global longitudinal strain of more than 15%, accordingly to the 2022 ESC Cardio-Oncology guideline [13]. All transthoracic echocardiogram was performed by Phillips EPIQ7 machine. Cardiac MRI diagnosis of myocarditis was based on the updated Lake Louise Criteria [14]. Two cardiology fellows (DHL and SC) reviewed patient’s clinical progress at least three times a week for surveillance of cardiac events (daily progress note of primary team or consultation to cardio-oncology service). In cases of cardiac events or CRS grade 2 or above, these events were adjudicated and managed by the cardio-oncology attending. After CAR-T infusion, the presence of CRS and adverse cardiac events were monitored and recorded during index hospitalization and up to 12 months after hospitalization. We defined adverse cardiac events as new onset CTCRD or heart failure, tachyarrhythmia (atrial fibrillation/ flutter, ventricular tachycardia), bradyarrhythmia (sinus bradycardia, 2nd degree or higher AV block), acute coronary syndrome, or cardiovascular death. Arrhythmias were detected through abnormality of heart rate on routine vital sign or when patients were symptomatic. After index hospitalization, patients are seen in cellular therapy outpatient clinic typically twice a week until day 30 after CAR-T, and then at 3, 6, and 12 months after CAR-T. Cardiovascular deaths were adjudicated through retrospective chart review only. In cases of cardiac events during hospitalization or outpatient, patients were seen in cardio-oncology clinic.

### Prospective observational study of inflammatory cytokine

Separate to this study, a subset of patients were enrolled in a prospective study on inflammatory biomarkers in subjects who underwent CAR-T (USF IRB Pro00021733). The study was open to all patients who underwent commercial CAR-T and all subjects who provided written informed consent was included in the study, as previously described [15]. The current study utilized the serum samples of these subjects. Serum inflammatory cytokines were measured at baseline before lymphodepletion and daily during hospitalization (N = 53). The number of samples varied as the duration of hospitalization was different between patients. The inflammatory cytokines quantified were interleukin (IL)-2, IL-6, IL-15, interferon-gamma (IFN-γ), tumor necrosis factor-alpha (TNF-α), granulocyte-macrophage colony-stimulating factor (GM-CSF), and angiopoietin 1 & 2. The serum was analyzed through the ELLA automated SimplePlex Immunoassay system (Protein Simple, USA) as previously described [15]. A two-fold serum dilution at a total of 50µL per each well was used. Each cartridge tests 32 samples in triplicates and tests four cytokines. The number of serial samples differ between patients as duration of hospitalization was different. The maximum level was designated as the highest level measured serially for individual cytokines after initiation of CAR-T. We present the baseline prior to lymphodepletion and maximum level of serum cytokine level, as previously described [15].

### Retrospective review

Clinical data were extracted retrospectively from electronic medical records, including demographics, baseline cardiac and oncologic risk factors, and baseline laboratory and transthoracic echocardiogram. Cardio-oncologists reviewed all electrocardiograms and transthoracic echocardiograms. Echocardiogram data was extracted through clinical reports. LV dimensions were calculated from parasternal long axis. LV ejection fraction was calculated through method-of-disc (MOD) method. LA volume was calculated through MOD method, indexed to body surface area. Data were collected and managed using REDCap electronic data management system hosted at Moffitt Cancer Center [16].

### Statistical analysis

Continuous variables were presented as mean ± standard deviation or median with interquartile range, depending on the normality of data using the Shapiro-Wilk normality test. Continuous variables were compared between groups using the student’s t-test or nonparametric comparison (Mann-Whitney test) depending on the normality of the data. Categorical data were compared using a chi-square test. Univariate logistic regression analyses were performed to report odds ratio and 90% confidence intervals (95% C.I.) of factors associated with cardiac events. Kaplan Meier survival analysis and a log-rank test were performed to determine differences in median overall survival durations of patients with and without cardiac events. A two-tailed p-value ≤0.05 was considered statistically significant. Statistical analysis was performed using R Software version 4.0.4 and GraphPad Prism (version 9) [17].

## Results

### Characteristics of cardiac events during CAR-T

Ninety patients were included in this study. The cohort’s median age was 68 years, with 61.1% being male **(**Table 1**)**. Eleven patients developed adverse cardiac events (12.2%). One patient developed cardiomyopathy with reduced left ventricular ejection fraction and was later diagnosed with acute myocarditis based on cardiac MRI. Ten patients (11.1%) developed atrial fibrillation, one of whom had a history of atrial fibrillation prior to CAR-T therapy. A total of 26 patients (28.9%) underwent follow-up TTE at a median of 10 days [interquartile range of 6–23 days] after CAR-T.

**Table 1:** Baseline Clinical Characteristics Based on Cardiac Events

Variables	Cardiac Events (N = 11)	No Cardiac Events (N = 79)	p value
Age at CAR-T	77.0 [69.5;79.0]	66.0 [56.5;73.0]	**0.002**
Gender			0.241
Male	9 (81.8%)	46 (58.2%)	
Female	2 (18.2%)	33 (41.8%)	
Ethnicity			0.747
White	11 (100.0%)	75 (94.9%)	
Black	0 (0.0%)	3 (3.8%)	
Other	0 (0.0%)	1 (1.3%)	
Cancer Type			0.518
DLBCL	5 (45.5%)	49 (62.0%)	
TFL	1 (9.1%)	7 (8.9%)	
B-ALL	0 (0.0%)	3 (3.8%)	
MCL	5 (45.5%)	20 (25.3%)	
Type of CAR-T			0.514
Yescarta	4 (36.4%)	36 (45.6%)	
Kymriah	2 (18.2%)	19 (24.1%)	
Tecartus	5 (45.5%)	20 (25.3%)	
Breyanzi	0 (0.0%)	4 (5.1%)	
Heart Rate (bpm)	79.7 ± 9.7	86.4 ± 16.2	0.186
Systolic BP (mmHg)	122.9 ± 18.9	116.0 ± 15.7	0.185
Diastolic BP (mmHg)	71.2 ± 12.9	71.7 ± 8.4	0.905
Creatinine (mg/dL)	0.9 [ 0.8; 1.1]	0.7 [ 0.6; 0.8]	**0.007**
CRP (mg/dL)	2.6 [ 0.7; 5.9]	2.2 [ 0.8; 5.8]	0.935
Hemoglobin (g/dL)	9.5 [ 7.9;10.1]	9.3 [ 8.2;10.8]	0.596
Platelet count (in 10^3^/uL)	94.0 [83.5;124.0]	143.0 [87.0;179.0]	0.148
Troponin I (ng/mL)	0.013 [0.010;0.036]	0.010 [0.010;0.011]	0.055
BNP (pg/mL)	59.5 [25.7;113.0]	51.0 [18.0;145.5]	0.775
LV Ejection Fraction (%)	55.0 [55.0;60.0]	60.0 [55.0;60.0]	0.502
MV E Velocity (cm/s)	70.9 ± 15.2	67.2 ± 18.2	0.527
MV A Velocity (cm/s)	75.0 [68.5;86.5]	74.0 [63.5;87.5]	0.815
MV E/A Ratio	0.9 [ 0.8; 1.1]	0.9 [ 0.7; 1.1]	0.494
Average E/e’	10.8 [ 8.9;15.9]	9.2 [ 7.0;11.4]	0.082
LV Diameter in Diastole (cm)	5.1 ± 0.5	4.7 ± 0.6	0.054
LV Diameter in Systole (cm)	3.4 ± 0.5	3.1 ± 0.5	0.12
IV Septum Thickness (cm)	1.0 ± 0.1	0.9 ± 0.2	0.123
Posterior Wall Thickness (cm)	1.0 ± 0.2	1.0 ± 0.2	0.169
Aortic Root Diameter (cm)	3.1 ± 0.5	2.9 ± 0.4	0.283
Indexed LA Volume (mL/m^2^)	23.9 [19.5;25.8]	16.9 [13.8;23.4]	**0.042**
Global Longitudinal Strain (%)	-17.0 ± 2.4	-17.7 ± 3.5	0.647

Data presented as Median [ Interquartile range], mean ± standard deviation, or number (percentage) depending on the type and normality of data

Abbreviations: CAR-T: chimeric antigen-receptor T cell, LBCL: large B- cell lymphoma, FL: follicular lymphoma, B-ALL: B-cell acute lymphoid leukemia, MCL: mantle cell lymphoma, MV: mitral valve, LV: left ventricle, IV: interventricular, LA: left atrium

### Clinical characteristics during CAR-T

Baseline cardiac comorbidities were not significantly different in the cardiac events group, including prior heart failure, coronary artery disease, atrial arrhythmia, and chronic kidney disease **(**Table 2**)**. Patients in the cardiac events group were older (median 77 vs. 66 years; p = 0.002), had higher baseline creatinine (median 0.9 vs. 0.7 mg/dL; p = 0.007) and a larger indexed left atrium volume (median 23.9mL/m^2^ vs. 16.9mL/m^2^, p = 0.042), when compared to those who did not develop cardiac events **(**Table 1**).** After CAR-T therapy, the incidence of grade 2 or above CRS was similar in both groups (36.4% vs. 31.6%; p = 1.000) **(**Table 3**)**. There was no difference in the rate of treatment of tocilizumab between those who did or did not develop cardiac events (72.7% vs. 53.2%; p = 0.368). There were eleven deaths (12.2%) one-year post-CAR-T. Of those, nine deaths were due to cancer progression, one due to CRS, and one due to pneumonia. The patients with cardiac events did not exhibit worse survival compared with patients without cardiac events (Log-rank, p = 0.200).

**Table 2:** Baseline Cardiac Comorbidities Based on Cardiac Events

Variables	Cardiac Events (N = 11)	No Cardiac Events (N = 79)	p- Value
Hypertension	6 (54.5%)	51 (64.6%)	0.755
Hyperlipidemia	4 (36.4%)	34 (43.0%)	0.925
Diabetes Mellitus	1 (9.1%)	13 (16.5%)	0.851
CAD (Revascularized)	0 (0.0%)	8 (10.1%)	0.589
CHF/ Cardiomyopathy	2 (18.2%)	7 (8.9%)	0.668
Stroke or TIA	0 (0.0%)	2 (2.5%)	1
COPD	1 (9.1%)	1 (1.3%)	0.577
OSA	0 (0.0%)	5 (6.3%)	0.876
PVD	1 (9.1%)	1 (1.3%)	0.577
CKD Stage I-III	1 (9.1%)	0 (0.0%)	0.246
CKD Stage IV-V	0 (0.0%)	1 (1.3%)	1
Atrial Arrhythmia	1 (9.1%)	11 (13.9%)	1
Ventricular Arrhythmia	0 (0.0%)	1 (1.3%)	1
Smoking History			0.973
Past	4 (36.4%)	26 (32.9%)	
Never	6 (54.5%)	45 (57.0%)	
Current	1 (9.1%)	8 (10.1%)	

Data presented as number (percentage)

Abbreviations: CAD: Coronary artery disease, CHF: chronic heart failure, TIA: transient ischemic attack, COPD: chronic obstructive pulmonary disease, OSA: obstructive sleep apnea, PVD: peripheral vascular disease, CKD: chronic kidney disease

**Table 3:** Post-CAR-T Clinical Manifestation Based on Cardiac Events

Variables	Cardiac Events (N = 11)	No Cardiac Events (N = 79)	P Value
Maximum CRS Grade			
0	0 (0.0%)	14 (17.7%)	
1	5 (45.5%)	40 (50.6%)	
2	5 (45.5%)	24 (30.4%)	
3	1 (9.1%)	0 (0.0%)	
4	0 (0.0%)	0 (0.0%)	
5	0 (0.0%)	1 (1.3%)	
CRS Classification			0.247
Grade 0/1 CRS	5 (45.5%)	54 (68.4%)	
Grade 2 or above CRS	6 (54.5%)	25 (31.6%)	
Tocilizumab Administration			0.368
Yes	8 (72.7%)	42 (53.2%)	
No	3 (27.3%)	37 (46.8%)	
Peak CRP (mg/dL)	11.7 [ 8.5;15.7]	13.0 [ 6.2;17.2]	1
Troponin Level, Day 5 (ng/mL)	0.010 [0.010;0.010]	0.010 [0.010;0.017]	0.307
BNP Level, Day 5 (pg/mL)	125.0 [86.0;268.0]	63.0 [21.0;124.0]	**0.019**

Data are presented as number (percentage %) or median with [interquartile range] or mean ± standard deviation

Abbreviations: CRS: cytokine release syndrome, CRP: c-reactive protein

### Cardiac biomarkers during CAR-T

The baseline troponin-I (0.013 vs. 0.010ng/mL; p = 0.055) and BNP (59.5 vs. 51.0pg/mL; p = 0.775) were similar between the two groups (N = 88, two patients without cardiac biomarkers). After CAR-T therapy, the cardiac events group had a higher BNP level on day 5 (median of 125.0 ng/dL vs. 63.0 ng/dL, p = 0.019). Day 5 troponin-I levels during hospitalization were not different between the two groups. **(**Table 3**)**. There was no association between cardiac biomarkers performed at baseline or day 5 post-CAR-T and cardiac events in univariate logistic regression analysis. Of the 31 patients who developed CRS grade 2 or above, post-CAR-T TTE was performed in 17 patients (54.8%).

### Inflammatory cytokines during CAR-T

**Fig. 2:**
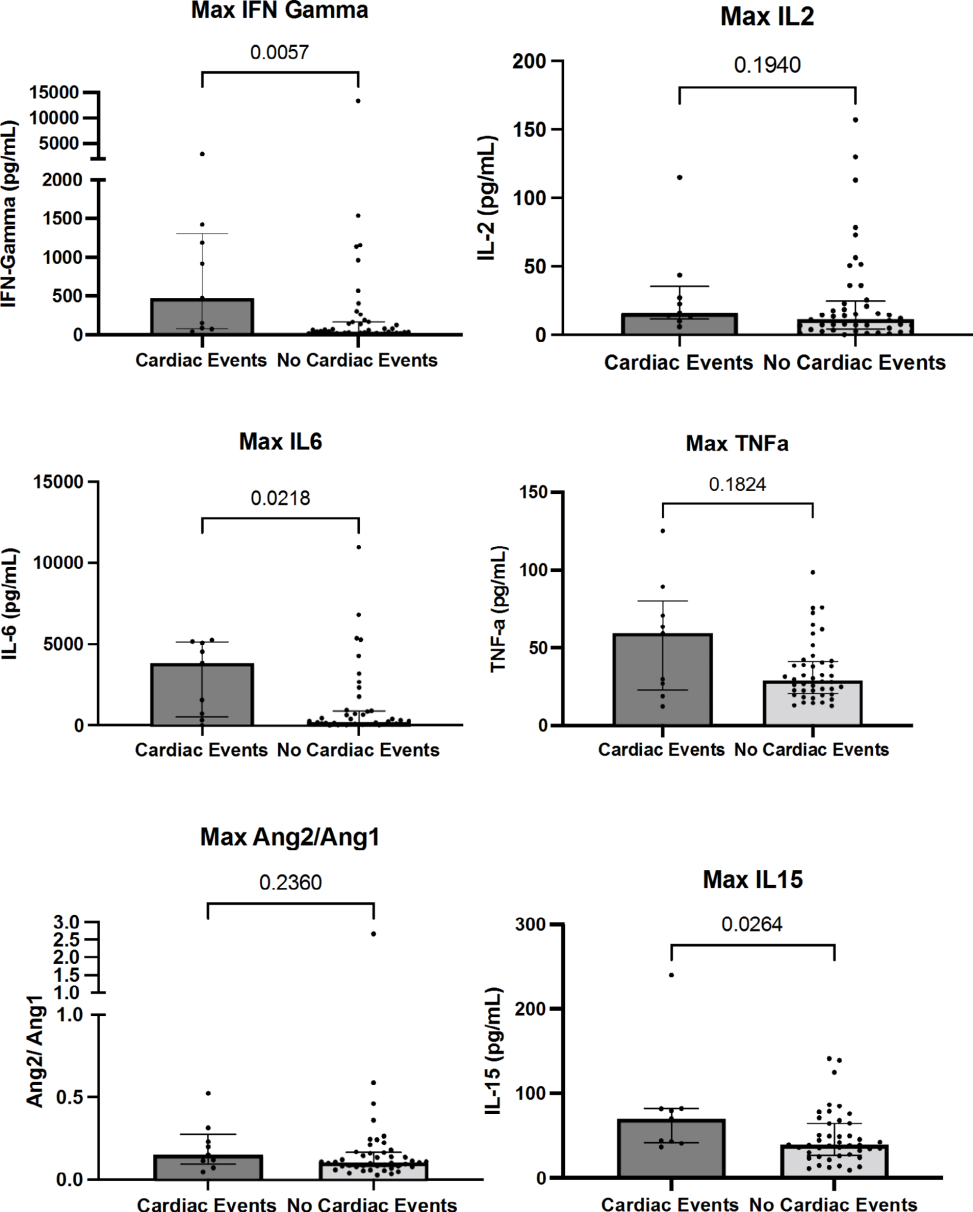
Differences in inflammation-related cytokines in adverse cardiac events Bar graph with scatter plot of the maximum level of individual cytokines during index hospitalization are summarized in the y-axis of individual graphs. The levels indicate the mean with standard error of mean (error bar) with individual values as dots. The maximum level after CAR-T injection is compared between the group with cardiac events versus no cardiac events. These cytokine levels were compared with non-parametric comparison. P values are indicated in each graph.

**Fig. 3:**
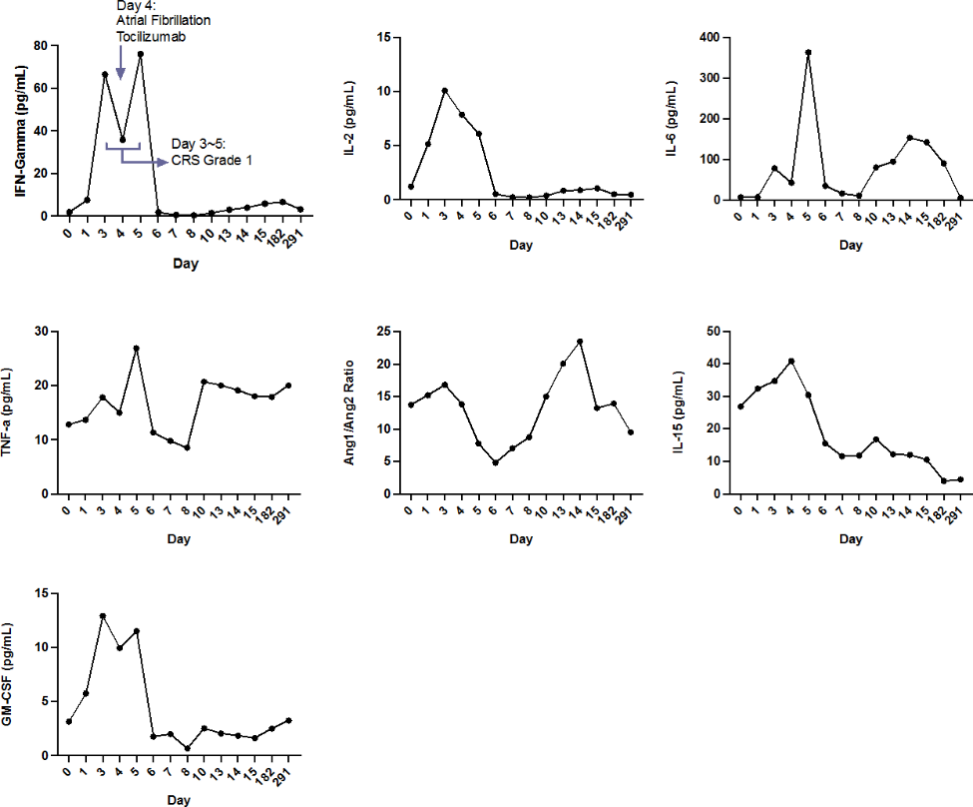
Representative serial inflammatory biomarker on a patient who developed atrial fibrillation An example of patient with serial inflammatory cytokines are measured at baseline and serially after CAR-T therapy. Patient developed cytokine release syndrome (CRS) grade 1 starting day 3 until day 5. On day 4, patient developed atrial fibrillation, and received tocilizumab for persistent CRS.

A total of fifty-three patients (58.9% of the total cohort) had serum testing of inflammatory cytokines at baseline and daily during hospitalization. Of the total eleven patients who developed cardiac events, nine had inflammatory cytokines tested. Baseline clinical characteristics, post-CAR-T clinical events and laboratory values were similar in patients who did and those who did not have inflammatory cytokine data (**Supplementary Tables 1–3**). Inflammatory cytokine levels are summarized in Fig. 2. Baseline levels of inflammatory cytokines were not different in those who subsequently developed cardiac events. However, peak IFN-γ (474.0 vs. 48.8pg/mL; p = 0.006), IL-6 (3855.0 vs. 254.0pg/mL; p = 0.021) and IL-15 (70.2 vs. 39.2pg/mL; p = 0.026) were higher in patients who developed cardiac events. We did not detect differences in peak IL-2, TNF-α and angiopoietin 2/1 between the two groups. A representative patient with serial inflammatory cytokines is summarized in Fig. 3. Pro-inflammatory cytokines, including IFN-γ, IL-6 peaked during CRS and cardiac events occurred in close proximity after CRS onset.

## Discussion

In the current study, clinical characteristics and cardiac/inflammatory biomarkers related to adverse cardiac events after CD19-directed CAR-T cell therapy in B-cell malignancies were assessed. In the cohort of 90 patients, the incidence of overall adverse cardiac events after CAR-T was similar to other studies (12.2%), with 11.1% of patients experiencing atrial arrhythmia and one developing cardiomyopathy (1.1%). We identified that mostly clinical factors (such as age, baseline creatinine, left atrial volume) were associated with cardiac events. However, cardiac (Troponin I and BNP) and inflammatory cytokine levels were not associated with cardiac events after CAR-T.

Notably, our cohort had differences in the type of cardiac events compared to prior studies [6–8, 11]. While the overall incidence of adverse cardiac events after CAR-T in our study was similar to that reported in prior studies (10–15%), the rate of atrial arrhythmia was 5–10% during CAR-T therapy, which is in line with our finding of 11.1% [18]. However, unlike other studies where the incidence of cardiomyopathy ranged from 5 to 15%, the current study had only one patient who developed cardiomyopathy (1.1%) during the follow-up period.

In our study, the baseline clinical factors, such as age and baseline creatinine were associated with adverse cardiac events similar to prior literature [6, 7, 11, 12]. In addition, unlike prior studies, our cohort had predominance of atrial arrhythmia in the types of cardiac events. This can be partially explained by the mildly larger left atrial volume in the cardiac events group.

We investigated cardiac biomarkers five days after CAR-T to assess the role of cardiac biomarkers in cardiotoxicities. Day 5 troponin-I was not elevated in those who developed cardiac events. Only one in ten patients who developed atrial fibrillation had elevated day 5 troponin I. Troponin-I is a traditional biomarker associated with new-onset cardiomyopathy, myocarditis, arrhythmia and cardiovascular death in cancer patients [19]. In pediatric/ adolescent patients receiving CAR-T therapy, daily troponin levels were measured in CRS (Grade 2 or higher) or cardiac events (heart failure, arrhythmias) [20]. Of those, 30.7% had abnormal troponin levels and those who had abnormal troponin levels all had cardiac dysfunction with decreased LVEF on echocardiogram. Although prior literature collectively suggests a role for serial measurements of troponin for early detection of cardiomyopathy, our cohort did not have interval troponin changes. This is likely due to lack of cardiomyopathy cases. In fact, our one case of cardiomyopathy secondary to myocarditis had only mildly elevated troponin I level at day 5 (0.032) from baseline level (0.010).

In contrast, there was only a tendency for day 5 BNP levels association with cardiac events. The accentuated BNP level after CAR-T in the setting of cardiac events may be a reflection of baseline comorbidities (history of cardiomyopathy, higher E/e’ ratio) although not statistically significant due to low number. This may lead to sensitization to hemodynamic stress in cardiac events such as atrial fibrillation. Prior observational studies have shown inconsistent results regarding BNP levels and their association with cardiotoxicity [21, 22]. While it is not fully understood, the discrepancy of cardiac biomarkers could be explained by physiologic changes (unfavorable hemodynamic profile with altered diastolic function) associated with atrial fibrillation before myocardial injury [23]. A recent report showed similar results where the authors prospectively analyzed high-sensitivity troponin-T and NT-pro BNP levels at baseline, and days 1, 7 and 21 after CAR-T [24]. It was also found that troponin-T did not change during CAR-T therapy, but NT-proBNP levels were elevated on day 7. However, the authors did not correlate the finding with cardiotoxicity.

Of note, the current cohort had a lower incidence of high-grade CRS. Only two patients (2.2%) developed high-grade CRS with CRS grade 3 and 5 and no CRS grade 4. This is in comparison to an 8–22% incidence of high-grade CRS 3 or above in clinical trials and real-world commercial CAR-T use [25, 26]. Although speculative, a shift in the management of CRS may play a role. In the cohort, nearly half of the patients who developed CRS grade 1 received tocilizumab. In fact, a shorter duration between CRS onset and tocilizumab administration was associated with reduced cardiac events [6]. Therefore, one hypothesis for a lower incidence of certain adverse cardiac events, especially the CTRCD, could be early use of tocilizumab by prevention of higher-grade CRS. However, further investigations are warranted to elucidate this relationship.

Contrary to prior literature, adverse cardiac events did not occur more frequently in higher-grade CRS. To that end, we performed a comprehensive analysis of inflammatory cytokines to see if pro-inflammatory status plays a role in the pathophysiology of cardiac events. Several pro-inflammatory cytokines (IL-6, IFN-γ and IL-15) were higher in the cardiac events group. This is in line with prior studies showing elevated IL-6 and IL-15 in patients with atrial fibrillation [27, 28]. However, the differences in the level of different cytokines were not significant enough to be used for clinical purpose. Furthermore, not all pro-inflammatory cytokines were elevated in the setting of cardiac events (IL-2 and TNF-α), likely due to limited number of samples. It does, however, suggest that the cardiac events are indeed related to a pro-inflammatory condition related to CRS.

## Study limitations

There are several limitations to the current study. First, the study was a retrospective review of cardiac events. The method of detecting arrhythmia with abnormal heart rate or symptoms may underestimate the incidence of atrial fibrillation. However, the incidence of AF are comparable to other studies. To our knowledge, there is no study published that has performed continuous telemetry monitoring for all CAR-T patients. For other cardiac events, a close collaboration between the cardio-oncology and cellular immunotherapy teams was developed to evaluate for cardiotoxicities. Therefore, the chances of missing cardiotoxicity after CAR-T were low. Second, we have not performed daily measurements of cardiac biomarkers, which could have missed pre-clinical cardiac injury. Next, the current exploratory study had a small sample size and lower incidence of adverse cardiac events which did not allow for multivariate analysis. The strength of our study includes the measurement of inflammatory cytokines in the setting of CAR-T related cardiac events. Until now, the association between CRS and cardiac events were the only evidence for inflammation-induced cardiac events. Also, our study population represents the relatively recent management changes of CRS with earlier and aggressive use of tocilizumab. Although speculative, this may have changed the incidences of more severe cardiac events such as cardiomyopathy/ heart failure. Further studies are needed with larger cohort size to ascertain this hypothesis.

In conclusion, adverse cardiac events, predominantly atrial fibrillation, occur commonly after CAR-T. The changes in serial inflammatory cytokine after CAR-T in the setting of adverse cardiac events suggests pro-inflammation as a pathophysiology and warrant further investigation.

## Electronic supplementary material

Below is the link to the electronic supplementary material.

Supplementary Material 1

## Data Availability

Data sharing is not applicable to this article as no datasets were generated or analyzed during the current study.

## Abbreviations

CAR-T: Chimeric antigen receptor T-cell
CRS: Cytokine release syndrome
LBCL: Large B- cell lymphoma
FL: Follicular lymphoma
B-ALL: B-cell acute lymphoid leukemia
MCL: Mantle cell lymphoma
CRP: C-reactive protein
BNP: B-type natriuretic peptide
ASTCT: American Society for Transplantation and Cellular Therapy
IL: Interleukin
GM-CSF: Granulocyte-macrophage colony-stimulating factor
TNF-α: Tumor necrosis factor alpha
IFN-γ: Interferon gamma
