# Supplementary material for: Cardiac and inflammatory biomarker differences in adverse cardiac events after chimeric antigen receptor T-Cell therapy: an exploratory study
Source: Cardiooncology. 2023 Apr 1;9:18. doi: 10.1186/s40959-023-00170-5 (PMC10067156; doi:10.1186/s40959-023-00170-5)
Supplement: Supplementary file 1 — Supplementary Material 1 [file 40959_2023_170_MOESM1_ESM.docx]

**Supplementary Table 1 Baseline Clinical Characteristics Based on Assessment of Inflammatory Cytokine Measurements**

| **Variables** | **No Inflammatory Cytokine** | **Inflammatory Cytokine** | **P Value** |
| --- | --- | --- | --- |
|  | **(N=37)** | **(N=53)** |  |
| Age at CAR-T | 68.0 [60.0;73.0] | 68.0 [58.0;76.0] | 0.761 |
| Gender |  |  | 1 |
| Male | 23 (62.2%) | 32 (60.4%) |  |
| Female | 14 (37.8%) | 21 (39.6%) |  |
| Ethnicity |  |  | 0.079 |
| White | 34 (91.9%) | 52 (98.1%) |  |
| Black | 3 (8.1%) | 0 (0.0%) |  |
| Other | 0 (0.0%) | 1 (1.9%) |  |
| Cancer Type |  |  | 0.243 |
| DLBCL | 22 (59.5%) | 32 (60.4%) |  |
| TFL | 2 (5.4%) | 6 (11.3%) |  |
| B-ALL | 0 (0.0%) | 3 (5.7%) |  |
| MCL | 13 (35.1%) | 12 (22.6%) |  |
| Type of CAR-T |  |  | 0.532 |
| Yescarta | 16 (43.2%) | 24 (45.3%) |  |
| Kymriah | 7 (18.9%) | 14 (26.4%) |  |
| Tecartus | 13 (35.1%) | 12 (22.6%) |  |
| Breyanzi | 1 (2.7%) | 3 (5.7%) |  |
| Heart Rate (bpm) | 83.2 ± 14.7 | 87.3 ± 16.3 | 0.234 |
| Systolic BP (mmHg) | 116.7 ± 16.6 | 116.9 ± 16.0 | 0.949 |
| Diastolic BP (mmHg) | 71.3 ± 8.3 | 71.8 ± 9.6 | 0.784 |
| Creatinine (mg/dL) | 0.7 [ 0.5; 0.9] | 0.7 [ 0.6; 0.9] | 0.669 |
| CRP (mg/dL) | 2.5 [ 0.7; 6.0] | 1.9 [ 0.9; 4.9] | 0.845 |
| Hemoglobin (g/dL) | 9.6 [ 8.1;10.9] | 9.2 [ 8.3;10.7] | 0.993 |
| Platelet count (in 10^3^/uL) | 136.0 [60.0;168.0] | 142.5 [94.0;215.5] | 0.093 |
| Troponin I (ng/mL) | 0.0 [ 0.0; 0.0] | 0.0 [ 0.0; 0.0] | 0.195 |
| BNP (pg/mL) | 85.0 [16.0;194.0] | 40.5 [20.0;112.0] | 0.224 |
| LV Ejection Fraction (%) | 60.0 [55.0;60.0] | 55.0 [55.0;60.0] | 0.287 |
| MV E Velocity (cm/s) | 69.3 ± 19.7 | 66.6 ± 16.5 | 0.481 |
| MV A Velocity (cm/s) | 79.0 [65.0;92.0] | 72.0 [64.0;86.0] | 0.175 |
| MV E/A Ratio | 0.8 [ 0.7; 1.0] | 0.9 [ 0.7; 1.1] | 0.597 |
| Average E/e' | 9.6 [ 7.5;11.3] | 8.9 [ 7.1;11.9] | 0.894 |
| LV Diameter in Diastole (cm) | 4.8 ± 0.7 | 4.7 ± 0.5 | 0.204 |
| LV Diameter in Systole (cm) | 3.2 ± 0.6 | 3.1 ± 0.4 | 0.159 |
| IV Septum Thickness (cm) | 0.9 ± 0.2 | 0.9 ± 0.2 | 0.906 |
| Posterior Wall Thickness (cm) | 0.9 ± 0.2 | 1.0 ± 0.2 | 0.389 |
| Aortic Root Diameter (cm) | 2.9 ± 0.5 | 3.0 ± 0.4 | 0.555 |
| Indexed LA Volume (mL/m^2^) | 33.0 [27.0;55.0] | 32.0 [25.0;44.0] | 0.301 |
| Global Longitudinal Strain (%) | -17.2 ± 2.8 | -17.8 ± 3.8 | 0.509 |

**Supplemental Table 2. Baseline Cardiac Comorbidities Based on Assessment of Inflammatory Cytokine Measurements**

| **Variables** | **No Inflammatory Cytokine** | **Inflammatory Cytokine** | **P Value** |
| --- | --- | --- | --- |
|  | **(N=37)** | **(N=53)** |  |
| Hypertension | 25 (67.6%) | 32 (60.4%) | 0.635 |
| Hyperlipidemia | 14 (37.8%) | 24 (45.3%) | 0.626 |
| Diabetes Mellitus | 5 (13.5%) | 9 (17.0%) | 0.88 |
| CAD (Revascularized) | 4 (10.8%) | 4 (7.5%) | 0.874 |
| CHF/ Cardiomyopathy | 5 (13.5%) | 4 (7.5%) | 0.568 |
| Stroke or TIA | 0 (0.0%) | 2 (3.8%) | 0.64 |
| COPD | 1 (2.7%) | 1 (1.9%) | 1 |
| OSA | 4 (10.8%) | 1 (1.9%) | 0.177 |
| PVD | 0 (0.0%) | 2 (3.8%) | 0.64 |
| CKD Stage I-III | 0 (0.0%) | 1 (1.9%) | 1 |
| CKD Stage IV-V | 1 (2.7%) | 0 (0.0%) | 0.856 |
| Atrial Arrhythmia | 6 (16.2%) | 6 (11.3%) | 0.721 |
| Ventricular Arrhythmia | 0 (0.0%) | 1 (1.9%) | 1 |
| Smoking History |  |  | 0.648 |
| Past | 12 (32.4%) | 18 (34.0%) |  |
| Never | 20 (54.1%) | 31 (58.5%) |  |
| Current | 5 (13.5%) | 4 (7.5%) |  |

**Supplemental Table 3 Post-CAR-T Clinical Manifestation Based on Assessment of Inflammatory Cytokine Measurements**

| **Variable** | **No Inflammatory Cytokine** | **Inflammatory Cytokine** | **P Value** |
| --- | --- | --- | --- |
|  | **(N=37)** | **(N=53)** |  |
| Maximum CRS Grade |  |  |  |
| 0 | 5 (13.5%) | 9 (17.0%) |  |
| 1 | 21 (56.8%) | 24 (45.3%) |  |
| 2 | 10 (27.0%) | 19 (35.8%) |  |
| 3 | 0 (0.0%) | 1 (1.9%) |  |
| 4 | 0 (0.0%) | 0 (0.0%) |  |
| 5 | 1 (2.7%) | 0 (0.0%) |  |
| CRS Classification |  |  | 0.575 |
| Grade 0/1 CRS | 26 (70.3%) | 33 (62.3%) |  |
| Grade 2 or above CRS | 11 (29.7%) | 20 (37.7%) |  |
| Tocilizumab Given? |  |  | 0.981 |
| No | 17 (45.9%) | 23 (43.4%) |  |
| Yes | 20 (54.1%) | 30 (56.6%) |  |
| Peak CRP (mg/dL) | 13.4 [ 6.4;17.0] | 13.0 [ 7.2;16.5] | 0.661 |
| Troponin Level, Day 5 (ng/mL) | 0.0 [ 0.0; 0.0] | 0.0 [ 0.0; 0.0] | 0.63 |
| BNP Level, Day 5 (pg/mL) | 90.0 [22.0;123.2] | 67.1 [29.0;151.0] | 0.868 |
